# Knowledge of undisclosed corporate authorship (“ghostwriting”) reduces the perceived credibility of antidepressant research: a randomized vignette study with experienced nurses

**DOI:** 10.1186/1756-0500-5-490

**Published:** 2012-09-05

**Authors:** Jeffrey R Lacasse, Jonathan Leo, Andrea N Cimino, Kristen F Bean, Melissa Del-Colle

**Affiliations:** 1 School of Social Work, College of Public Programs, Arizona State University, Phoenix, Arizona, USA; 2 Lincoln Memorial University- DeBusk College of Osteopathic Medicine, Harrogate, TN, USA

## Abstract

**Background:**

There is much concern regarding undisclosed corporate authorship (“ghostwriting”) in the peer-reviewed medical literature. However, there are no studies of how disclosure of ghostwriting alone impacts the perceived credibility of research results.

**Findings:**

We conducted a randomized vignette study with experienced nurses (n = 67), using a fictional study of antidepressant medication. The vignette described a randomized controlled trial and gave efficacy and adverse effect rates. Participants were randomly assigned to one of two authorship conditions, either (a) traditional authorship (n = 35) or (b) ghostwritten paper (n = 32), and then completed a perceived credibility scale. Our primary hypothesis was that the median perceived credibility score total would be lower in the group assigned to the ghostwritten paper. Our secondary hypotheses were that participants randomized to the ghostwritten condition would be less likely to (a) recommend the medication, and (b) want the psychiatrist in the vignette as their own clinician. We also asked respondents to estimate efficacy and adverse effect rates for the medication.

There was a statistically significant difference in perceived credibility among those assigned to the ghostwriting condition. This amounted to a difference of 9.0 points on the 35-point perceived credibility scale as tested through the Mann–Whitney U test. There was no statistically significant difference between groups in terms of recommending the medication, wanting the featured clinician as their own, or in estimates of efficacy or adverse effects (*p >* .05 for all such comparisons).

**Conclusion:**

In this study, disclosure of ghostwriting resulted in lower perceived credibility ratings.

## Findings

### Background

There is much concern regarding conflicts-of-interest [1,2] and authorship within the peer-reviewed medical literature, particularly in pharmaceutical industry-funded randomized controlled trials of therapeutics [3-7]. This has largely been sparked by a growing body of evidence from medico-legal cases demonstrating the prevalence of “ghostwriting,” [8] an undisclosed conflict-of-interest in which a pharmaceutical company employee or subcontractor co-authors a study but is not listed on the authorship byline [9]. It is now common for evidence of ghostwriting to be available on the internet for widely used blockbuster medications [10-12]. Increasingly, clinicians are likely to encounter revelations of ghostwriting for established treatments both within medical journals [11-14] and in the general media, such as the *New York Times*[15,16].

As reported in both a prominent randomized controlled trial and a recent systematic review of the literature, financial conflicts-of-interest (COIs) are known to reduce the perceived credibility of research [17,18]. However, little is known regarding how practicing clinicians perceive ghostwritten research. To our knowledge, there has been only one study directly related to this issue [19]. This was a study of 50 hospital-based clinicians randomly assigned to receive one of two research vignettes, one in which the author had a financial COI and in which the article had been ghostwritten, and the other in which there was no COI and traditional authorship. The research results in the COI group were much less believed by clinicians (Cohen’s *d =* 1.4), suggesting that disclosure of relatively common COIs had a clinically significant impact on their view of the research. The major limitation of the study was that the COI conditions were bundled (there was no “ghostwriting only” condition) so that the impact of financial COI or ghostwriting could not be identified separately. We therefore conducted a follow-up study examining the impact of ghostwriting alone on perceived credibility.

### Methods

Two vignettes modified slightly from the original study were used [19]. Both vignettes contained an identical description of a fictional study of a new antidepressant (“Serovux”) for adult use. The vignettes described the study sample, methods, and results; the conclusion claimed that Serovux was a safe and effective treatment, utilizing language from a well-known antidepressant study [10,20]. One vignette described the research as having been ghostwritten (ghostwriting condition), while the other described traditional academic authorship (non-ghostwriting condition). Other than modifying the reported COI and changing the clinical population from children to adults, no major changes were made to the vignettes used in the original experiment. We opted not to change vignette content substantially because we had found evidence of face validity in our earlier study; ghostwriting experts agreed that these vignettes described a ghostwriting incident “similar to those known to have occurred in real life” and agreed that a clinician reading the scientific literature would be “likely to come across studies that were generated in a manner similar to that described” [19]. The vignettes are available as Additional files (see Additional file 1: Vignette 1.pdf and Additional file 2: Vignette 2.pdf).

Our primary outcome measure was perceived credibility of the results, measured through a 7-item Likert scale that asked respondents to rate how truthful, accurate, credible, honest and sincere the research vignette was. Although this scale has not been formally validated in terms of psychometrics, it was derived from items within the existing perceived credibility instrument literature, and this instrument was found to be reliable (Cronbach’s alpha = 0.95) in a previous study [19]. We measured two types of secondary outcomes. First, two dichotomous questions answered in a yes/no format, [1] “If I was having problems with depression, I would like to have Dr. Harvey [The name of the psychiatrist featured in the vignette] as my psychiatrist” and “If a friend you cared about was severely depressed, would you recommend Serovux?”. Second, we asked respondents to estimate the percentage of patients who (a) benefitted significantly from Serovux, and (b) had significant side effects from Serovux.

Our primary hypothesis was that the median perceived credibility score total would be lower in the group assigned to the ghostwriting condition. Our secondary hypotheses were that participants randomized to the ghostwritten condition would be less likely to (a) recommend the medication, and (b) want the psychiatrist in the vignette as their own clinician. We also speculated that when asked to estimated efficacy and adverse effect rates, participants in the ghostwriting group would discount the reported rate of efficacy and inflate the reported rate of adverse effects.

We performed power analysis using G*Power 3.04 [21]. Guided by our previous results, we calculated that we needed 35 participants per group to detect a large difference in perceived credibility (Cohen’s *d =* 0.8) with 95% power. Data analysis was performed using PASW version 18.0 (SPSS, Inc, Chicago, IL, USA) and Minitab version 15.1.0.0 (Mini-tab Inc., State College, Pennsylvania, USA). Our data analysis strategy consisted of comparing total credibility scores by group using the Mann–Whitney U test, appropriate for use with ordinal data that is not normally distributed, as well as contingency table analysis for the dichotomous variables. Given our small sample size, 95% confidence intervals were to be reported to define the precision of our point estimates. Secondarily, we also planned to describe mean total credibility scores across groups and calculate effect size in the form of Cohen’s *d*.

Participants were recruited from two sources. First, data were collected from students in a graduate-level nurse practitioner course taking place at a large Southwestern University in a major metropolitan area. Using a random number generator (http://www.random.org), the sequence of the printed vignettes and attached instrumentation was randomized, which were then handed out to students in the classroom setting. The researcher distributing the materials was blind to their content (i.e., he did not know which vignette was distributed to each nurse). All students in the class were invited to voluntarily participate. Second, an invitation to take an on-line version of the survey was distributed to community nurses though a local email listerv. The survey was placed online using SurveyGizmo survey software, and randomization was automated. The title of the project was “Perceptions of Biomedical Research” and participants were not aware that the goal of the project was to examine the impact of ghostwriting on perceived credibility. Institutional review board approval was granted by the Arizona State University Office of Research Integrity and Assurance, and each participant provided informed consent.

### Results

Sixty-seven nurses participated in this study, meaning that our study was underpowered according to our pre-study power analysis. Fifty-seven of the sixty-one nurses registered for the course participated in this study, for a response rate of 93.44%. We did not track whether the nursing students who did not participate (n = 4) refused participation or were simply not in attendance for that class session. An additional ten nurses completed the online version of the survey.

There were no missing data for the perceived credibility items. The Cronbach’s alpha for the perceived credibility scale used in the present study was 0.91. A principal components analysis (PCA) extracted only one component thus establishing that our 5-question credibility scale was a unidimensional measure of credibility. The two questions which asked about the efficacy/side effect rate of Serovux had 7.5% and 9.0% missing data, respectively, while the remaining variables all had minimal missing data (<5%). Given the small amount of missing data, we performed complete-case analyses [22].

The majority of participants were female, Caucasian, and were experienced clinicians (see Table 1). Respondents randomized to the non-ghostwriting vignette had a mean credibility score of 22.00 ±5.87, while those assigned to the ghostwriting vignette had a mean credibility score of 15.37 ±7.85. This results in a difference in perceived credibility of 6.63 points, a Cohen’s *d* of 0.96. Since the data did not meet the assumption of normality necessary for parametric tests and the credibility scale is ordinal, we tested the difference using the Mann–Whitney U statistic [23]. This resulted in a difference between medians of 9.0 points (95% CI = 4.0 - 12.0; see Table 2).

**Table 1:** Participant demographics by vignette group*

**Characteristic**	**Non-Ghostwriting Vignette (n = 35)**	**Ghostwriting Vignette (n = 32)**
Age, years**†**	36.97 ± 11.26	36.00 ± 10.25
Female gender, No. (%)	35 (100)	30 (93.75)
Caucasian, No. (%)	24 (68.57)	24 (75.0)
Bachelors in Nursing, No. (%)**‡**	23 (65.71)	26 (81.25)
Clinical experience, years**†**	12.51 ± 9.82	11.04 ± 9.84
Clinical experience in mental health, years**†**	7.26 ± 10.12	6.31 ± 9.42

* Does not include cases with missing data (2 cases for race/ethnicity, 1 case for clinical experience, and 3 cases for clinical experience in mental health).

**†** Values are presented as means ± Standard Deviation.

**‡** Refers to highest degree earned.

**Table 2:** Credibility ratings by ghostwriting condition

**Outcome Variable**	**No Ghostwriting (n = 35)**		**Ghostwriting (n = 32)**		P-value*	Difference of Medians (95% CI)*
	**Mean ± SD**	**Median**	**Mean ± SD**	**Median**		
Truthful	4.54 ±1.25	5.00	3.28 ±1.78	3.00	.003	
Accurate	4.23 ±1.21	4.00	3.72 ±1.76	3.50	.144	
Credible	4.14 ±1.26	4.00	2.84 ±1.78	2.50	.001	
Honest	4.46 ±1.42	4.00	2.53 ±1.90	2.00	<.001	
Sincere	4.63 ±1.37	5.00	3.00 ±1.95	2.00	<.001	
Overall Credibility Scale Total**†**	22.00 ±5.87	23.00	15.37 ±7.85	13.00	<.001	9.00 (4.00 – 12.00)

*As calculated using the Mann–Whitney U test.

**†** Out of an achievable scale total of 35 points.

There were no statistically significant differences across groups on any of the remaining variables. Respondents randomized to the ghostwriting condition were less likely to want the psychiatrist in the vignette as their own clinician (OR = 0.34, 95% CI = 0.10 - 1.24), or to recommend the antidepressant (“Serovux”) featured in the vignette (OR = 0.44, 95% CI = 0.13 - 1.49), but these results were not statistically significant. The mean estimated efficacy rate for Serovux was 53.53% ±14.12 in the non-ghostwriting condition and 47.50% ±24.59 in the ghostwriting condition, and the mean estimated side effect rate was 11.85% ±7.86 in the non-ghostwriting condition and 13.24% ±10.65 in the ghostwriting condition, with identical median values across both vignette conditions (*p >* .05 for all these comparisons).

We had combined two subsamples for analysis, nurses taking a graduate-level class (n = 57) and nurses participating in a local email listserv for community nurses (n = 10). As a separate analysis, we excluded the nurses reached through the email listserv (n = 10) and re-analyzed the data. The results were essentially the same (i.e., <0.5 points difference in overall perceived credibility ratings).

### Discussion

Experienced nurses (n = 67) who read a fictional antidepressant study found the vignette less credible when informed that the research was ghostwritten. The difference was statistically significant (*p <* .001), consisting of a 9-point difference (95% CI, 4.00 -12.00) between medians on a 35-point scale. There was a difference of 6.63 points in mean scores, corresponding to a Cohen’s *d* of 0.96. By normal social science standards, this is considered a large effect size [24]. To our knowledge, this is the first published study to find an impact of ghostwriting alone on perceived credibility.

This finding has several potential implications. The previous study [19] used vignettes reporting a pediatric antidepressant study, raising the possibility that respondents were reacting to the issue of pediatric psychiatric prescribing, a controversial issue in some quarters. The present study reached similar findings while reporting an adult antidepressant study, suggesting that the results of these studies are not dependent on the population described. These results also may inform the ongoing debate regarding the ability of clinicians to engage in evidence-based practice within an environment of less-than-ideal transparency in authorship, selective reporting of data, and so forth. In short, activities such as ghostwriting and selective reporting have ramifications downstream for the clinicians who rely on such data to practice ethically and scientifically [25-28]. Although this is somewhat speculative, the large effect size found in this study could be interpreted as indicating that practicing clinicians consider ghost authorship an important issue worthy of attention and regulation. Finally, legal scholars are now exploring the issue of ghostwriting, and our findings may have ramifications for this body of literature as well [29,30].

However, our finding that disclosure of ghostwriting is associated with lower perceived credibility also raises many questions that cannot be answered by the present study. As suggested by two anonymous reviewers, these include: Why do the nurses perceive ghostwritten research as less credible? Are nurses aware of the problems that ghostwriting presents to the integrity of science, or are they reacting to some other factor? Is the primary issue the challenge to evidence-based medicine, a reaction to a deceptive practice, or hostility to the pharmaceutical industry? Are nurses aware of the International Committee of Medical Journal Editors (ICMJE) authorship standards, which were violated in this study? Is it reasonable to expect any seasoned health professional to form an opinion of a medication or clinician based on one short vignette? These are all research questions worthy of further research, and many are probably best addressed through qualitative inquiry.

After completing two research studies on ghostwriting, one potential explanation for the finding of reduced credibility has emerged, albeit subjectively, anecdotally, and speculatively. During our data collection, we encountered anecdotal evidence suggesting that practicing clinicians are not generally aware of the practice of ghostwriting (e.g., from written comments on the instrumentation and verbal comments spoken to the investigators as subjects returned their instruments). If this is broadly true, a portion of the reduced credibility may be due to a disconnect between how clinicians envision research and authorship and the process described in the ghostwriting vignette condition. Future investigations on this topic might consider this hypothesis.

Although the nurses in our study found ghostwritten research less credible, the other variables we examined had smaller effect sizes. Subjects randomized to the ghostwriting condition were less likely to want the psychiatrist in the vignette as their own clinician or to recommend the antidepressant for a depressed friend, but this difference was not statistically significant. This is partially a result of low statistical power for these variables, as we powered the study to examine our primary outcome, which we assumed had a large effect size. However, there were other nuances in these data. For both of these questions, only 10/34 (29.41%) participants in the non-ghostwriting group endorsed the psychiatrist and antidepressant portrayed in the vignette. We consider this a low level of endorsement given that the non-ghostwriting vignette presented a rigorous clinical trial similar to many well-cited reports of real-world antidepressant research. This skepticism may reflect growing public dissatisfaction with the pharmaceutical industry [31], knowledge of the contemporary debate regarding antidepressant medications [32-35], or unknown factors.

However, these findings are also suggestive of the complexities involved in moving from perceived credibility (a fairly straightforward outcome measure) to decision making. Although subjects randomized to the ghostwriting condition had lower credibility scores, they did not substantially discount the efficacy of the medication nor inflate the rate of adverse effects. In other words, they found the ghostwritten study less credible; but when asked to act or report on the data given to them, many arguably answered as if they did indeed believe the results. This may reflect defensive decision making on the part of prescribers [36], or the use of heuristics [37] in decision making.

Our study has several limitations that should shape interpretation of the results. Most of the nurses in the study were pursuing an advanced degree and thus may not be representative of practicing nurses in general. Only nurses from one geographic locale were surveyed. Also, this was a small study only capable of capturing large effect sizes. Future studies should utilize larger sample sizes as well as include participants from multiple geographic locales, as well as other health professionals (e.g., bioethicists, psychiatrists, etc.).

The majority of our participants were taking a graduate-level class on evidence-based nursing, which may have had an impact on our results. Graduate programs generally strive to teach research within the context of critical thinking. It is possible that the students in our sample were more critically minded than the average clinician, a hypothesis congruent with other observations of graduate students [38,39]. However, previous vignette research [19] examining practicing clinicians’ (not graduate students) perceptions has found lower credibility ratings associated with COI.

In conclusion, we find that disclosure of ghostwriting alone, without other accompanying COI, is sufficient to lower the perceived credibility of psychiatric research to an important degree among professional nurses. This finding is congruent with our previous study finding that a package of relatively common COI, including ghostwriting, also lowered the perceived credibility of psychiatric research. Moving forward, we suggest that qualitative research exploring the thought processes behind participants’ ratings and treatment decisions would be informative.

## Abbreviations

COI: Conflict-of-interest; CI: Confidence interval; OR: Odds ratio; PCA: Principal component analysis.

## Competing interests

JRL and JL are members of Healthy Skepticism, an international non-profit organization dedicated to reducing harm from misleading drug promotion. JRL serves on the Scientific Advisory Council of the Foundation for Excellence in Mental Health Care.

## Authors’ contributions

Conceived the study: JRL. Collected the data: JRL KFB. Analyzed the data and wrote first draft: JRL. Participated in writing the paper: ANC JL JRL KFB MDC. All authors read and approved final version of manuscript: ANC JL JRL KFB MDC.

## Supplementary Material

Additional file 1 **Vignette 1.Traditional authorship, no ghostwriting.** Click here for file

Additional file 2 **Vignette 2.Ghostwriting.** Click here for file
