# Supplementary material for: Knowledge of undisclosed corporate authorship (“ghostwriting”) reduces the perceived credibility of antidepressant research: a randomized vignette study with experienced nurses
Source: BMC Res Notes. 2012 Sep 5;5:490. doi: 10.1186/1756-0500-5-490 (PMC3532151; doi:10.1186/1756-0500-5-490)
Supplement: Additional file 2 — Vignette 2.Ghostwriting. [file 1756-0500-5-490-S2.pdf]

Vignette 2- Ghostwriting

Dr. Harvey is a researcher and professor of psychiatry at Northern University, an esteemed academic institution. Dr. Harvey is a very prestigious researcher.

Dr. Harvey recently participated in a study of Serovux (an antidepressant). Dr. Harvey treated some of the patients in this study.

This study was written up by Biopsychiatric Pharmaceuticals, Inc., the company that makes Serovux. The marketing department did not want to list company employees as the only authors, because readers may notice that all of the authors work directly for the company that makes Serovux. So, Biopsychiatric Pharmaceuticals, Inc. sent Dr. Harvey a pre-written manuscript, authored by employees of the company, and asked Dr. Harvey to be listed as first author. Although he did not write the manuscript, Dr. Harvey agreed to be listed as the primary author of the study.

Dr. Harvey submitted the manuscript to a prominent medical journal, as if he had written it himself. The journal reviewed it and agreed to publish it.

Speaking at a press conference about the study, Dr. Harvey said: "Clinical depression is a major health problem, and we need to find effective treatments. So, we did a randomized trial of Serovux- the most rigorous kind of experiment you can do.

We took 200 depressed adults, and we randomly prescribed them either Serovux or sugar pills (placebo). The sugar pills looked just like Serovux pills. Neither the patients nor the doctors knew which pill they were taking.

We followed the patients for eight weeks, and then assessed them several times. We did clinical interviews to see if they were still depressed, and if so, how severe it was. We used the Hamilton Depression Inventory (HAM-D), which is a scientifically valid way of assessing depression.

We found that 62% of the patients who were taking Serovux were rated as "much improved" or "very much improved," compared to 34% of patients taking placebo. 46% of patients taking Serovux had complete remission of their depression, as did 21% of patients taking placebo.

We found that side effects were rare- less than 10% of the patients experienced any significant side effects.

We conclude that Serovux is a well-tolerated and effective treatment for clinical depression."
